# *Calomys callosus* chronically infected by *Toxoplasma gondii* clonal type II strain and reinfected by Brazilian strains is not able to prevent vertical transmission

**DOI:** 10.3389/fmicb.2015.00181

**Published:** 2015-03-10

**Authors:** Priscila S. Franco, Neide M. da Silva, Bellisa de Freitas Barbosa, Angelica de Oliveira Gomes, Francesca Ietta, E. K. Shwab, Chunlei Su, José R. Mineo, Eloisa A. V. Ferro

**Affiliations:** ^1^Laboratory of Immunophysiology of Reproduction, Department of Histology and Embryology, Federal University of Uberlândia, Uberlândia, Brazil; ^2^Laboratory of Immunopathology, Institute of Biomedical Sciences, Federal University of Uberlândia, Uberlândia, Brazil; ^3^Department of Life Sciences, University of Siena, Siena, Italy; ^4^Department of Microbiology, The University of Tennessee, Knoxville, TN, USA; ^5^Laboratory of Immunoparasitology, Department of Immunology, Microbiology and Parasitology, Federal University of Uberlândia, Uberlândia, Brazil

**Keywords:** *Toxoplasma gondii*, congenital toxoplasmosis, parasite genotypes, Brazilian strains, reinfection

## Abstract

Considering that *Toxoplasma gondii* has shown high genetic diversity in Brazil, the aim of this study was to determine whether *Calomys callosus* chronically infected by the ME-49 strain might be susceptible to reinfection by these Brazilian strains, including vertical transmission of the parasite. Survival curves were analyzed in non-pregnant females chronically infected with ME-49 and reinfected with the TgChBrUD1 or TgChBrUD2 strain, and vertical transmission was analyzed after reinfection of pregnant females with these same strains. On the 19th day of pregnancy (dop), placentas, uteri, fetuses, liver, spleen, and lung were processed for detection of the parasite. Blood samples were collected for humoral and cellular immune response analyses. All non-pregnant females survived after reinfection and no changes were observed in body weight and morbidity scores. In pregnant females, parasites were detected in the placentas of ME-49 chronically infected females and reinfected females, but were only detected in the fetuses of reinfected females. TgChBrUD2 reinfected females showed more impaired pregnancy outcomes, presenting higher numbers of animals with fetal loss and a higher resorption rate, in parallel with higher levels of pro-inflammatory cytokines and IgG2a subclass antibodies. Vertical transmission resulting from chronic infection of immunocompetent *C. callosus* is considered a rare event, being attributed instead to either reactivation or reinfection. That is, the pregnancy may be responsible for reactivation of the latent infection or the reinfection may promote *T. gondii* vertical transmission. Our results clearly demonstrate that, during pregnancy, protection against *T. gondii* can be breached after reinfection with parasites belonging to different genotypes, particularly when non-clonal strains are involved in this process and in this case the reinfection promoted vertical transmission of both type II and Brazilian *T. gondii* strains.

## INTRODUCTION

*Toxoplasma gondii*, the causative agent of toxoplasmosis, is an obligate intracellular protozoan parasite belonging to the eukaryotic phylum Apicomplexa that can infect all warm-blooded animals (Sullivan and Jeffers, 2012). This parasite has a complex life cycle consisting of a sexual cycle in its feline definitive host and an asexual cycle in its intermediate host. Intermediate hosts, including humans, can be infected after the ingestion of raw or undercooked meat containing tissue cysts or after consumption of food or water contaminated with oocysts (Schlüter et al., 2014). *T. gondii* infects up to a third of the world’s population and the infection is generally asymptomatic among humans and animals with normal immunity, but it can cause a high level of morbidity and mortality in immunocompromised individuals (Schlüter et al., 2014). Moreover, *T. gondii* can also be transmitted from the mother to the fetus, resulting in abortion or fetal abnormalities (Carlier et al., 2012; Adams Waldorf and McAdams, 2013).

In congenital toxoplasmosis, transmission to the fetus occurs predominantly in women who acquire primary infection during pregnancy (Remington et al., 2011; Carlier et al., 2012). In immunocompetent mothers who have been infected with *T. gondii* before conception, it has been preconized that immune mechanisms prevent transmission of the infection to their fetuses (Bojar and Szymanska, 2010). However, acquired immunity due to *T. gondii* infection does not fully protect against severe consequences to the child, caused either by reactivation of a latent infection in pregnant women with immunocompromised status or by reinfection, especially if the parasite strain is non-clonal (Silveira et al., 2003; Elbez-Rubinstein et al., 2009). Both cellular and humoral components of the immune response play critical roles in resistance against *T. gondii* infection. Marked immunological modifications occur during pregnancy, which promote maternal tolerance to paternal alloantigens, leading to the successful implantation of the placenta and ensuring survival of the developing fetus. Increased hormone concentrations, i.e., progesterone, inhibit IL-12, TNF-α, and NO production by macrophages, increase IL-10 production by dendritic cells (DCs) and thereby dampen the development of strong Th1 cell responses. Consequently, pregnant women may be more susceptible to infection with *T. gondii* (Yarovinsky, 2014).

*Toxoplasma gondii* strains are genetically diverse. The genotype of the parasite has been implicated in disease severity (Elbez-Rubinstein et al., 2009; Wujcicka et al., 2014). *T. gondii* has a highly clonal genetic structure, with three major genetic types, I, II, and III, predominantly observed in North America and Europe (Howe and Sibley, 1995). On the other hand, an entirely distinct genotype pattern has been demonstrated in Central and South America, where an abundance of different strain types have been found (Pena et al., 2008; Khan et al., 2009; Su et al., 2012; Shwab et al., 2014). The type I strains, mostly found in South America, are highly virulent with a lethal dose 100 of ∼1 parasite (Khan et al., 2009), whereas type II and III strains are less virulent, with lethal doses 50 of ∼10^3^ and 10^5^ parasites, respectively (Saeij et al., 2006). Also, in South America both congenital and ocular toxoplasmosis are more prevalent compared to Europe and more often associated with severe symptoms. It has been proposed that this could be associated with non-clonal strains (not type I, II, or III), mainly those isolated from South America (Gilbert et al., 2008).

More recently, analysis of isolates from domestic animals in Brazil revealed over a 100 restriction fragment length polymorphism (RFLP) genotypes, with four of these isolates being considered common clonal lineages, designated types BrI, BrII, BrIII, and BrIV (Pena et al., 2008; Dubey et al., 2012). Analysis of mortality rates in infected mice indicated that Type BrI is highly virulent, Type BrIII is non-virulent, and Type BrII lineages are intermediately virulent (Pena et al., 2008). Two parasite strains were recently obtained from chickens in Uberlândia city, Minas Gerais, Brazil and they were named TgChBrUD1 and TgChBrUD2. The TgChBrUD1 strain exhibited ToxoDB PCR–RFLP genotype #11 (also known as type BrII) and the TgChBrUD2 strain exhibited ToxoDB PCR–RFLP genotype #6 (also known as type BrI and Africa 1).

The placenta is the primary interface between the fetus and mother and plays an important role in maintaining fetal development and growth by facilitating the transfer of substrates and participating in modulating the maternal immune response to prevent immunological rejection of the conceptus. In addition, the placenta produces hormones that alter maternal physiology during pregnancy and forms a barrier against the maternal immune system (Watson and Cross, 2005). During pregnancy, the important protective role of the placenta against maternal–fetal *T. gondii* transmission has been reported (Robert-Gangneux et al., 2011). Although the gross architecture of the human and mouse placentas differ somewhat in their details, their overall structures and the molecular mechanisms underlying placental development are thought to be quite similar. As a result, the mouse is increasingly used as a model for studying the essential elements of placental development (Watson and Cross, 2005).

*Calomys callosus* (Rodentia, Cricetidae), a characteristic rodent in central Brazil, has been described as a useful experimental model to study congenital toxoplasmosis (Ferro et al., 2002; Barbosa et al., 2007; Franco et al., 2011). Congenital toxoplasmosis studies have shown that *C. callosus* is resistant to *T. gondii* strain ME-49 and vertical transmission occurs only during the acute phase of infection (Ferro et al., 2002; Barbosa et al., 2007). A previous study in this model showed that after 60 days of infection (doi) vertical transmission is not observed (Barbosa et al., 2007). In addition, our recent study showed that congenital toxoplasmosis does not occur in females chronically infected with the moderately virulent ME-49 clonal strain and reinfected with the highly virulent *T. gondii* RH clonal strain (Franco et al., 2011). Also, we observed that *C. callosus* is susceptible to infection by *T. gondii* TgChBrUD1 or TgChBrUD2 strains, since these animals died during the acute phase and weight loss and several clinical signs were observed after infection (Franco et al., 2014).

Considering that a primary *T. gondii* infection in *C. callosus* can provide protective immunity against reinfection with the highly virulent RH strain and that reinfection with non-clonal strains can promote vertical transmission, the present study aimed to verify if *C. callosus* chronically infected with the ME-49 strain may be susceptible to reinfection by the *T. gondii* TgChBrUD1 or TgChBrUD2 strains and if such reinfection may cause vertical transmission of the parasite. Moreover, we investigated the *T. gondii* genotype in placentas and fetuses to verify the effect of the reinfection on the reactivation of a latent infection and immune protection after reinfection.

## MATERIALS AND METHODS

### ANIMALS

*Calomys callosus* were kept under standard conditions on a 12-h light, 12-h dark cycle in a temperature-controlled room (25 ± 2°C) with food and water *ad libitum* in the Animal Experimentation Center, Federal University of Uberlândia, Brazil. Animal experiments and procedures were conducted according to local institutional guidelines for ethics in animal experimentation and approved by the Ethical Committee for Animal Experimentation (Protocol: CEUA/UFU 049/11).

### PARASITE STRAINS

Cysts of the ME-49 strain were obtained from brains of *C. callosus* infected 30–45 days earlier with 20 cysts via the oral route as previously described (Barbosa et al., 2007). Briefly, brains were removed, homogenized, washed in sterile phosphate-buffered saline (PBS; pH 7.2) at 1000 × *g* for 10 min and cysts were counted under light microscopy for further experimental infection and strain maintenance. Tachyzoites of TgChBrUD1 and TgChBrUD2 strains were obtained initially from peritoneal exudates of previously infected Swiss mice and then maintained by serial passages in human fibroblast (HFF) cells. The cell culture-derived parasites were stained with 0.4% Trypan blue and counted in a hemocytometric chamber to determine the concentrations of viable parasites, which were to be used in experimental infection protocols.

### EXPERIMENTAL ANIMALS AND INFECTIONS

In the first set of experiments, *C. callosus* virgin females (*n* = 15), aged 2–3 months, were perorally infected with 20 cysts of *T. gondii* ME-49 strain. The females were randomly divided into three groups of five animals. After 60 doi, the reinfection was performed with an intraperitoneal inoculum of 100 tachyzoites of RH strain or TgChBrUD1 or TgChBrUD2 strains. Successful primary infection was determined by detection of specific antibodies in serum samples taken 5 days before reinfection. The females were monitored to evaluate body weight change, morbidity, and mortality for 25 days. Morbidity was assessed based on the clinical parameters as previously described (Bartley et al., 2006) with modifications: sleek/glossy coat, bright and active (score 0); hunched, starry stiff coat (score 1), reluctance to move (score 2).

In a second set of experiments, *C. callosus* virgin females (*n* = 28), aged 2–3 months, were randomly divided into four groups, as follows: Group 1 (Non-infected pregnant females), Group 2 (ME-49 chronically infected pregnant females), Group 3 (ME-49 chronically infected and TgChBrUD1 reinfected pregnant females), and Group 4 (ME-49 chronically infected and TgChBrUD2 reinfected pregnant females). Females of group 2, 3, and 4 were perorally infected with 20 cysts of ME-49 strain and after 60 doi, were mated with males and checked daily for the presence of a vaginal plug. The presence of a vaginal plug was considered as the first dop. The reinfection was carried out by an intraperitoneal inoculum of 100 tachyzoites of TgChBrUD1 or TgChBrUD2 strain for group 3 and 4, respectively at first dop. Blood samples were collected from all animals on the 55 doi before mating and reinfection for analysis of antibodies to *T. gondii*. The pregnant females were monitored to evaluate mortality and morbidity until the 19th dop, when animals were euthanized. The uteri were examined and the implantation sites were quantified. Normal and absorbed implantation sites were identified by visual observation. An implantation site with a shrunken placenta and a dissolved or discolored brown embryo was defined as an reabsorption site (Kusakabe et al., 2008). The fetal loss rate was calculated as the resorption sites by the total number of implantation sites (resorption plus normal implantation sites), as described previously (Joachim et al., 2001; Zenclussen et al., 2002). The uterus was collected only from reinfected females that presented resorption sites and no normal fetuses. Placentas, uteri and fetuses were collected for immunohistochemical, quantitative real-time PCR (qPCR) and RFLP–PCR assays. Placentas and fetuses were used for mouse bioassays. Different placentas and fetuses from the same female were used in these assays. In addition, liver, spleen and lung from pregnant females were collected for qPCR and RFLP–PCR assays. Blood samples were collected to determine the levels of IgG, IgG1, and IgG2a isotypes as well as the serum levels of Th1 and Th2 cytokines.

### IMMUNOHISTOCHEMICAL ASSAYS

For immunolocalization of the parasites in the tissue samples, formalin-fixed samples were dehydrated and embedded in paraffin. Tissue sections measuring 4 μm in thickness were placed on glass slides and processed, as previously described (Ferro et al., 2002). Briefly, samples were first incubated with 5% acetic acid to block endogenous alkaline phosphatase and then with 2% normal goat serum to block non-specific binding sites. Next, samples were incubated at 4°C overnight with mouse anti-*T. gondii* polyclonal serum (1:100), which was produced by our laboratory by infecting Swiss mice with ME-49 strain, and then with biotinylated goat anti-mouse IgG (1:600) (Sigma-Aldrich, St. Louis, MO, USA). The reaction was amplified by avidin-biotin-alkaline phosphatase system (ABC kit, PK-4000; Vector Laboratories, Inc., Burlingame, CA, USA) and developed with fast red-naphthol (Sigma). Samples were counterstained with Harris’s hematoxylin and examined under light microscopy.

### MOUSE BIOASSAY

Detection of *T. gondii* was evaluated by mouse bioassay as described elsewhere (Freyre et al., 2006; Barbosa et al., 2007). Placenta and fetal tissues (liver and brain) were homogenized in PBS and separately inoculated in Swiss mice by intraperitoneal route, in duplicate. Blood samples were collected at 35 days after inoculation and analyzed for seroconversion by ELISA and brains were collected for qPCR and RFLP–PCR assays.

### DNA EXTRACTION AND QUANTIFICATION OF *T. gondii* BY QUANTITATIVE REAL-TIME PCR

Total DNA was extracted from 20 mg of each tissues using Wizard^®^ Genomic DNA Purification Kit (Promega Co., Madison, WI, USA), according to the manufacturer’s instructions. The number of parasites in tissues was determined by qPCR of extracted DNA using a TaqMan probe targeting the ITS1 sequence (GenBank Accession# AY143141). The primers for PCR amplification were ITS1-Fx: AGCGAAGGGGCTCAATTTCT and ITS1-Rx: TGAAATAACGGTGTGGGAAA, which amplified a 117 bp sequence. The ITS1 probe was 6-FAM/CGTGTCTCTGTT-GGGATACTGATTTCCAGG/BHQ-1, with the 5′ end labeled with FAM and the 3′ end labeled with Black Hole Quencher-1 (BHQ-1; Eurofins Scientific, Longmont, CO, USA; Hill et al., 2012). The qPCR reaction had a total volume of 23 μl containing 17.74 μl of H_2_O, 2.5 μl of 10 × PCR buffer + MgCl_2_, 2.0 μl of 2.5 mM dNTP, 0.13 μl of 50 mM ITS1-Fx and ITS1-Rx primers, 0.2 μl of 50 mM ITS1 probe and 0.3 μl of 5 U μl Faststart Taq DNA polymerase (Roche Applied Science, Indianapolis, IN, USA). Two microliters of purified DNA was added as template and PCR reaction mix was transferred to 96 wells plate. The reaction was carried out using a iQ^™^ 5 Multicolor Real-Time PCR Detection System (Bio-Rad, Hercules, CA, USA) with the following conditions: 94°C for 60 s, then 45 cycles of 92°C for 15 s, 52°C for 30 s, and 72°C for 40 s. The threshold cycle (Ct) value for each sample was compared to the standard control (10^3^ to 10^7^ parasites/ml) and the relative parasite concentration was analyzed.

### MULTILOCUS RFLP–PCR GENOTYPING OF *T. gondii*

The genotyping of strains was determined by RFLP–PCR as described previously (Su et al., 2010). Briefly, multiplex PCR was carried out using a set of mixed external primers in a single reaction. The pre-amplification step consisted of 95°C for 4 min, followed by 30-cycle PCR at 94°C for 30 s, 55°C for 1 min, and 72°C for 2 min. A volume of 3 μl of the products served as template DNA for nested PCR with internal primers for each marker. The nested PCR amplification step consisted of 4 min at 95°C, followed by 35 cycles of 94°C for 30 s, 60°C for 60 s, and 72°C for 90 s. The nested PCR products were digested using the appropriate restriction endonucleases (Su et al., 2010). The restriction fragments were resolved in 2.5% agarose gel and observed under ultraviolet light. Images were digitally photographed for the interpretation of genotyping data. A negative control, without DNA, was included in each reaction mixture and GT1 (type I), PTG (type II), CTG (type III), TgCgCa1, MAS, TgCatBr5, TgCatBr1, and TgCatBr2 strains were used as positive controls.

### *T. gondii*-SOLUBLE ANTIGEN

*Toxoplasma gondii*-soluble tachyzoite antigen (STAg) was obtained as previously described (Mineo et al., 1980). Briefly, infected mouse peritoneal exudates (RH strain) were washed twice in PBS at 720 *g* for 10 min at 4°C. Parasite suspensions were adjusted to 1 × 10^8^ tachyzoites/mL, treated with protease inhibitors and then lysed by five freeze thaw cycles and further by sonication (six 60-Hz cycles for 1 min each) on ice. After centrifugation (10,000 *g*, 30 min, 4°C), supernatants were collected and filtered through a 0.2-μm membrane (Corning Incorporated Costar, New York, NY, USA). The protein concentration was determined by using the Lowry method (Lowry et al., 1951) and STAg aliquots were stored at –80°C.

### MEASUREMENT OF *T. gondii*-SPECIFIC TOTAL IgG, IgG1, AND IgG2a ISOTYPES

An indirect ELISA to detect serum IgG antibodies to *T. gondii* was carried out as previously described (Barbosa et al., 2007) in order to confirm preconceptional seroconversion of female *C. callosus*, as well as the seroconversion of Swiss mice inoculated in bioassay experiments as an indicator of *T. gondii* infection. In addition, IgG1 and IgG2a antibodies to *T. gondii* were carried out to evaluate the humoral immune response profile in pregnant reinfected *C. callosus* females. For detection of specific total IgG, low-binding polystyrene microtiter plates (Kartell SPA, Noviglio, Milan, Italy) were coated overnight at 4°C with *T. gondii* soluble antigen (10 μg/ml) in carbonate buffer 0.06 M (pH 9.6). After washing with PBS plus 0.05% Tween-20 (PBST), plates were incubated with serum samples (1:64) in 5% non-fat milk (PBS-TM) for 1 h at 37°C and subsequently with peroxidase-labeled goat anti-mouse IgG (1:1000, Sigma) in PBS-TM for 1 h at 37°C. For detection of specific IgG1 and IgG2a, high-binding polystyrene microtiter plates (Corning Incorporated Costar) were coated overnight at 4°C with *T. gondii* soluble antigen (10 μg/ml). Plates were blocked with 5% skim milk in PBS plus 0.05% Tween 20 (PBS-T) for 1 h. Serum samples were diluted 1:32 in 1% skim milk-PBS-T and incubated for 1 h at 37°C. IgG subclasses were detected with secondary goat anti-mouse IgG1 or anti-mouse IgG2a antibodies (1:1000, Sigma). After new washes, the reactions were developed with 0.03% hydrogen peroxide and 1 mg/ml o-phenylenediamine (OPD). The reaction was stopped with 2N H_2_SO_4_ and optical density (OD) was measured at 492 nm using a plate reader (Titertek Multiskan Plus, Flow Laboratories, Geneva, Switzerland). Results were expressed as ELISA index (EI) as follows: EI = OD sample/cut-off, where cut-off was established as mean OD values of negative control sera plus three standard deviations based on screening tests performed with negative and positive control sera. EI > 1.2 values were considered positive results.

### IFN-γ, TNF-α, IL-10, AND TGF-β MEASUREMENTS IN SERUM SAMPLES

The concentrations of cytokines were measured by sandwich ELISA. The IL-10 and TNF-α (OpTEIA, BD Bioscience, San Diego, CA, USA) and IFN-γ and TGF-β (Duoset R&D Systems, Minneapolis, MN, USA) cytokines were assayed according to instructions from the manufacturers. The concentrations of cytokines in serum samples from pregnant reinfected *C. callosus* females were calculated from a standard curve of each murine recombinant cytokine. The limit of detection in the ELISAs was 31.3 pg/ml (IFN-γ), 15.6 pg/ml (TNF-α), 31.3 pg/ml (IL-10), and 15.6 pg/ml (TGF-β). Intra-assay and inter-assay coefficients of variation were below 20% and 10%, respectively.

### STATISTICAL ANALYSIS

The data were analyzed using GraphPad Prism version 5.0 (GraphPad Software, San Diego, CA, USA). Data were expressed as mean ± S.D. of experimental groups. The Kaplan–Meier method was applied to estimate the percentage of *C. callosus* females surviving (survival rate) after reinfection and survival curves were compared using the Log-rank test. Comparisons of the differences among cytokines and parasitism among various experimental groups were performed by Mann–Whitney or Kruskal–Wallis tests with Dunn multiple comparison post-test. The antibody production comparison before and after reinfection, for each groups, was analyzed by the Mann–Whitney, whereas comparisons between groups were analyzed by Kruskal–Wallis. Differences were considered statistically significant when *P* < 0.05.

## RESULTS

### FEMALES CHRONICALLY INFECTED WITH ME-49 ARE ABLE TO SURVIVE AFTER REINFECTION WITH HIGHLY INFECTIVE PARASITE STRAINS

The infection outcomes in females chronically infected with ME-49 and reinfected with the TgChBrUD1 or TgChBrUD2 strains were investigated and the RH strain was used as control. It was observed that all females chronically infected with ME-49 survived after reinfection with RH, TgChBrUD1 or TgChBrUD2. In addition, no significant difference in body weight changes and morbidity scores was detected (data not shown).

### REINFECTION WITH BRAZILIAN STRAINS IS HARMFUL TO PREGNANCY AND REINFECTION WITH TgChBrUD2 RESULTED IN MORE FREQUENT IMPAIRED PREGNANCY OUTCOMES COMPARED WITH TgChBrUD1

**FIGURE 1:**
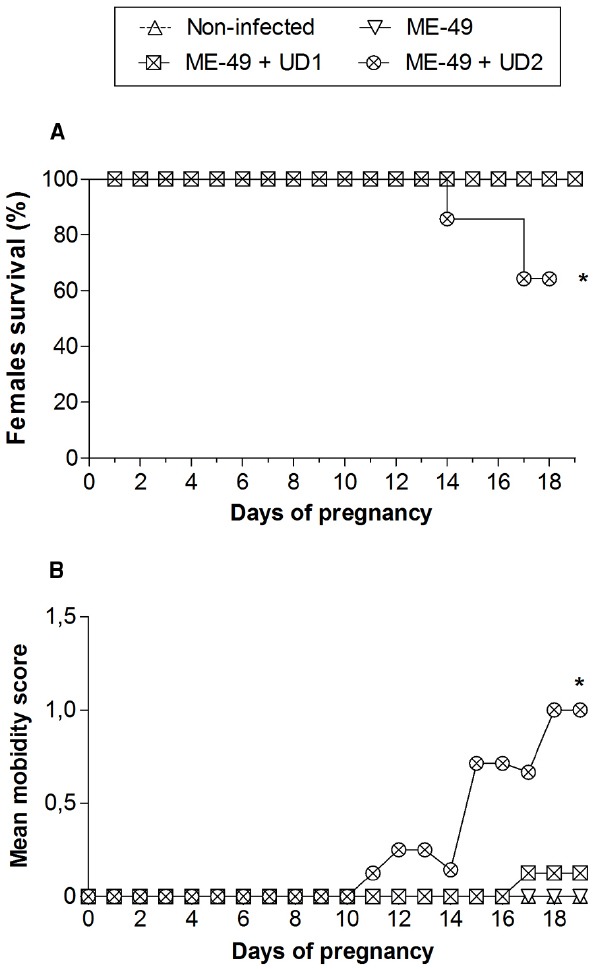
**(A)** Survival curves and **(B)** morbidity of non-infected *C. callosus* females, females chronically infected with ME-49 and females chronically infected with ME-49 and reinfected in the first day of pregnancy by TgChBrUD1 (UD1) or TgChBrUD2 (UD2) strains of *Toxoplasma gondii*. Females (n = 7 for each group) were monitored until the 19th day of pregnancy. *P < 0.05. **(A)** Log-rank testand. **(B)** Wilcoxon signed rank test.

The pregnancy in *C. callosus* females chronically infected with ME-49 was investigated after reinfection with *T. gondii* Brazilian strains on the first day of gestation. It was observed that all females from groups 1, 2, and 3 and five out of seven females from group 4 survived until the 19th dop (Table 1; Figure 1A). Comparison between groups showed survival rates significantly lower in group 4 (Figure 1A). Females from groups 1, 2, and 3 showed no change in morbidity scores (Figure 1B). On the other hand, females from group 4 started to be hunched with starry stiff coats around the 11th dop and demonstrated reluctance to move on the 15th day, showing higher morbidity scores (*P* < 0.05; Figure 1B).

**Table 1:** **Survival and pregnancy outcome for non-infected *C. callosus* females, females chronically infected with ME-49 and females chronically infected with ME-49 and reinfected in the first day of pregnancy by TgChBrUD1 (UD1) or TgChBrUD2 (UD2) strains of *Toxoplasma gondii***.

**Group**	**Strain**	**Female survival *n/N* (%)**	**Females with normal fetuses *n/N***	**Females with reabsorbed fetuses *n/N* (%)**	**Reabsorption sites/implantation^a^*n/N* (%)^b^**
1	Non-infected	7/7 (100)	7/7	3/7 (42.8)	3/28 (10.7)
2	ME-49	7/7 (100)	7/7	3/7 (42.8)	3/30 (10)
3	ME-49 + UD1	7/7 (100)	5/7	4/7 (57.1)	11/34 (32.3)
4	ME-49 + UD2	5/7 (71.2)	1/5	4/5 (80)	19/23 (82.6)

a Normal and reabsorbed implantation sites were identified by visual observations. An implantation site with a shrunk placenta and a dissolved or discolored brown embryo was defined as a reabsorption site. The n/N represent the total amount of reabsorption sites/implantation for all females for each group.

b The fetal loss rate was calculated as the reabsorption sites to the total number of implantation sites (reabsorption plus normal implantation sites).

On the 19th dop, all females in group 1 presented normal fetuses and three out of seven females presented reabsorbed fetuses (42.8%). A total of 28 implantation sites with three reabsorption sites were observed, showing fetal loss of 10.7% (Table 1). In group 2, all females presented normal fetuses and three out of seven females presented reabsorbed fetuses (42.8%). A total of thirty implantation sites with three reabsorption sites were observed, showing fetal loss of 10% (Table 1). In group 3, five out of seven females presented normal fetuses and four females presented reabsorbed fetuses (57.1%). Considering the four females who had reabsorbed fetuses, two of them showed only the implantation sites with signs of necrosis. A total of 34 implantation sites with eleven reabsorption sites were observed, showing fetal loss of 32.3%. In group 4, only one out of five females presented normal fetuses and four females presented reabsorbed fetuses (80%). Considering the four females who had reabsorbed fetuses, all of them showed the implantation sites with signs of necrosis. A total of 23 implantation sites with 19 reabsorption sites were observed, showing fetal loss of 82.6% (Table 1).

**FIGURE 2:**
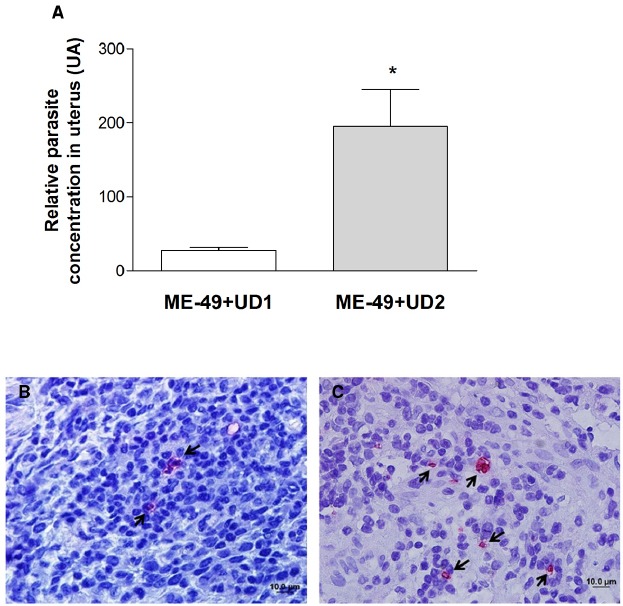
**(A)** Comparative parasite burden in the uterus tissue from TgChBrUD1 (UD1; n = 2) and TgChBrUD2 (UD2) reinfected pregnant females (n = 4). *P < 0.05 (Mann–Whitney test). **(B)** Representative photomicrographs of uterus tissue from TgChBrUD1 and **(C)** TgChBrUD2 reinfected pregnant female. Arrows indicate parasites inside the parasitophorous vacuoles.

Tissue parasitism was investigated by qPCR and immunohistochemistry in the uterus. All uteri from groups 3 and four presented parasites (Table 2) and TgChBrUD2 reinfected pregnant females had significantly higher uterine parasite loads in comparison to TgChBrUD1 reinfected pregnant females (Figure 2A). Immunohistochemical assays confirmed the presence of *T. gondii* in uterus (Figures 2B,C). The genotyping of parasites in two uteri from group 3 showed that one uterus presented the ME-49 strain and another one presented the TgChBrUD1 strain (Table 3). In group 4, the four uteri presented TgChBrUD2 strain parasites (Table 3).

**Table 2:** **Results of PCR assay for *C. callosus* females chronically infected with ME-49 and females chronically infected with ME-49 and reinfected in the first day of pregnancy by TgChBrUD1 (UD1) or TgChBrUD2 (UD2) strains of *Toxoplasma gondii*. PCR was also carried out in brain tissues from mouse in the bioassay**.

**Pregnant females**						
**Group**	**Strains**	**Positive PCR result in tissues from pregnant animals *n/N***			**Positive PCR result in brain (mouse bioassay) *n/N***	
		**Placentas**	**Fetuses**	**Uterus**	**Placentas**	**Fetuses**
1	–	NI	NI	NI	NI	NI
2	ME-49	4/7	0/7	NI	NI	NI
3	ME-49 + UD1	5/5	1/5	2/2	3/5 (2^†^)	1/5 (1^†^)
4	ME-49 + UD2	1/1	0/1	4/4	0/1 (1^†^)	1/1

† Animals died between 7 and 10 days after inoculum. NI, not investigated.

**Table 3:** **Genotyping of *Toxoplasma gondii* in tissues from placenta, fetus, uterus, liver, spleen, and lung from *C. callosus* females chronically infected with ME-49 and females chronically infected with ME-49 and reinfected in the first day of pregnancy by TgChBrUD1 (UD1) or TgChBrUD2 (UD2) strains of *Toxoplasma gondii*, as well as in the brain tissue from mouse bioassay. Data represent numbers of the samples and strain detected in each tissue**.

**Genotyping**									
**Group**	**Strain**	**Placenta**	**Fetus**	**Uterus**	**Liver**	**Spleen**	**Lung**	**Brains from mice bioassay**	
								**Placenta**	**Fetus**
1	–								
2	ME-49	(4) ME-49	x	x	(5) ME-49	(3) ME-49	(4) ME-49	NI	NI
3	ME-49 + UD1	(3) ME-49 (2) UD1	(1) UD1	(1) ME-49 (1) UD1	(4) UD1 (1) ME-49 and UD1	(1) ME-49 (4) UD1	(2) ME-49 (3) UD1	(2) ME-49	(1) ME-49
4	ME-49 + UD2	(1) UD2	x	(4) UD2	(5) UD2	(4) UD2	(4) UD2 (1) ME-49 and UD2	x	(1) UD2

x, absence of sample; NI, not investigated.

### THE ACQUIRED IMMUNE RESPONSE OF *Calomys callosus* FEMALES CHRONICALLY INFECTED WITH THE *T. gondii* ME-49 CLONAL STRAIN IS INSUFFICIENT TO PREVENT VERTICAL TRANSMISSION FOLLOWING REINFECTION WITH STRAINS FROM BRAZIL

**FIGURE 3:**
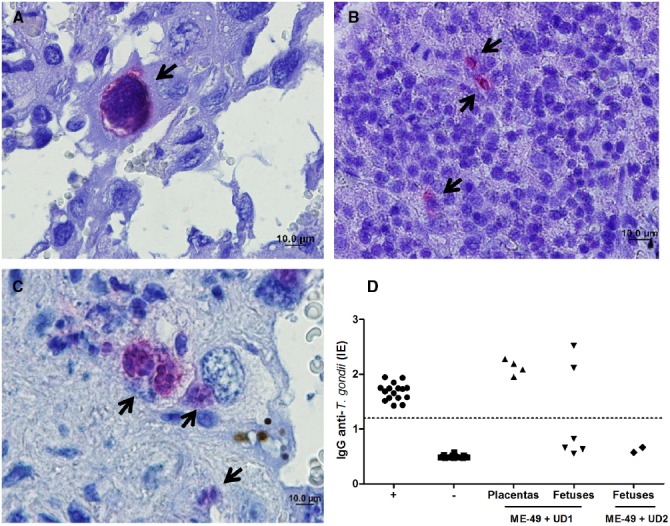
**Representative photomicrographs of (A) placenta and (B) fetus from TgChBrUD1 reinfected pregnant female and (C) placenta from TgChBrUD2 reinfected pregnant female.** Arrows indicate parasites inside the parasitophorous vacuoles. **(D)** IgG analysis of survival Swiss mice inoculated with placentas (n = 4) and fetuses (n = 6) tissues (liver and brain) from TgChBrUD1 (UD1) reinfected female or fetuses (n = 2) from TgChBrUD2 (UD2) reinfected female. Antibody levels were measured by ELISA and expressed as an ELISA index (EI).

Vertical transmission was evaluated by immunohistochemical assays, mouse bioassay and PCR. It was observed that females chronically infected with ME-49 did not transmit *T. gondii* vertically, while females chronically infected with ME-49 and reinfected with Brazilian strains were able to transmit vertically. Immunohistochemical results showed parasites in placenta and fetus tissues from group 3 and placenta from group 4 (Figures 3A–C).

The ELISA to detect IgG antibodies was carried out in serum from survival Swiss mice after 30 days after infection. *T. gondii*-specific IgG antibodies were detected in mice that were inoculated with placentas and fetuses from group 3 (Figure 3D). *T. gondii*-specific IgG antibodies not were detected in mice that were inoculated with fetuses from group 4 (Figure 3D).

The qPCR showed that *T. gondii*-DNA was detected in placentas from group 2 (*n* = 4), group 3 (*n* = 5), and group 4 (*n* = 1; Table 2). *T. gondii*-DNA was detected in fetus tissues from group 3 (*n* = 1). On the other hand, we did not observe *T. gondii*-DNA in fetal tissues from groups 2 and 4 (Table 2). The qPCR from brains of mice used in mouse bioassays showed that *T. gondii*-DNA was detected in mice inoculated with placenta tissues from three females of group 3. The mice inoculated with placenta tissues from the other two females of group 3 and from one female of group 4 died between 7 and 10 days after inoculation (Table 2). In addition, *T. gondii*-DNA was detected in mice inoculated with fetal tissues from groups 3 and 4 (Table 2). The mice inoculated with fetal tissues from one female of group 3 died between 7 and 10 days after inoculation (Table 2).

**FIGURE 4:**
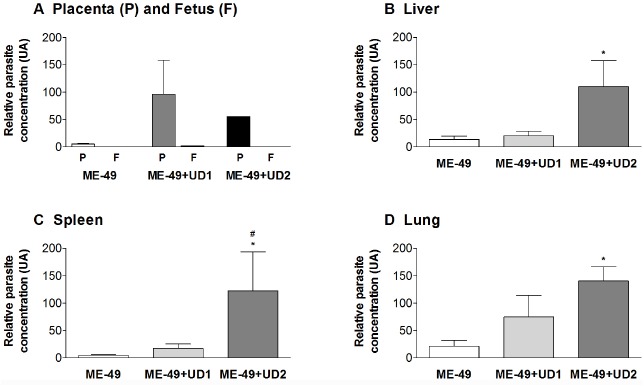
**Comparative parasite burden in the (A) placenta (P) and fetus (F), (B) liver, (C) spleen, and (D) lung tissues from ME-49 chronically infected pregnant females, TgChBrUD1 (UD1) and TgChBrUD2 (UD2) reinfected pregnant females (n = 7 for each group).** *Comparison between ME-49 chronically infected pregnant females and TgChBrUD1 or TgChBrUD2 reinfected pregnant females (Kruskal–Wallis test, P < 0.05). #Comparison between TgChBrUD1 and TgChBrUD2 reinfected pregnant females (Mann–Whitney test, P < 0.05).

Tissue parasitism was investigated and it was observed that ME-49 chronically infected females showed low parasite concentration in the placenta. On the other hand, all placentas from TgChBrUD1 and TgChBrUD2 reinfected females showed high parasite concentration, but with no significant difference (Figure 4A). When fetal tissues were analyzed, fetuses from TgChBrUD1 reinfected females showed low parasite concentration, while no parasite was detected for any fetuses from ME-49 chronically infected females and fetuses from TgChBrUD2 reinfected females (Figure 4A).

The genotyping of *T. gondii* was analyzed in qPCR positive samples. The ME-49 strain was observed in all placentas from group 2 (Table 3). In group 3, three females presented the ME-49 strain in the placenta, while two females and one female presented the TgChBrUD1 strain in the placenta and fetuses, respectively (Table 3). Group 4 presented the TgChBrUD2 strain in placentas (Table 3). In the brains from mice bioassays, the ME-49 strain was observed in placenta and fetus samples from TgChBrUD1 reinfected females (Table 3). The TgChBrUD2 strain was observed in fetus samples from TgChBrUD2 reinfected females (Table 3).

### TgChBrUD2 REINFECTED FEMALES PRESENTED HIGHER PARASITE BURDEN IN THE LIVER, SPLEEN AND LUNG

The qPCR results showed that TgChBrUD2 reinfected females had significantly higher parasite concentrations in liver, spleen and lung in comparison to ME-49 chronically infected females (Figures 4B–D) and significantly higher parasite concentration in spleen when compared with TgChBrUD1 reinfected females (Figure 4C). Comparison between organs in different groups of animals showed no significant difference between the ME-49 chronically infected females and the TgChBrUD1 and TgChBrUD2 reinfected female groups (data not shown). The genotyping of parasites in group 2 showed the ME-49 strain in liver (*n* = 5), spleen (*n* = 3), and lung (*n* = 4) (Table 3). In group 3, four females presented the TgChBrUD1 strain in the liver, while one female presented a mixed infection. One female presented the ME-49 strain and four females presented the TgChBrUD1 strain in the spleen. When the lung was analyzed, two females presented the ME-49 strain and three females presented the TgChBrUD1 strain (Table 3). Group 4 presented the TgChBrUD2 strain in liver (*n* = 5) and spleen (*n* = 4). Four females presented the TgChBrUD2 strain in the lung, while one female presented a mixed infection (Table 3).

### REINFECTED PREGNANT FEMALES SHOWED ELEVATED LEVELS OF IFN-γ, TNF-α, AND IL-10

**FIGURE 5:**
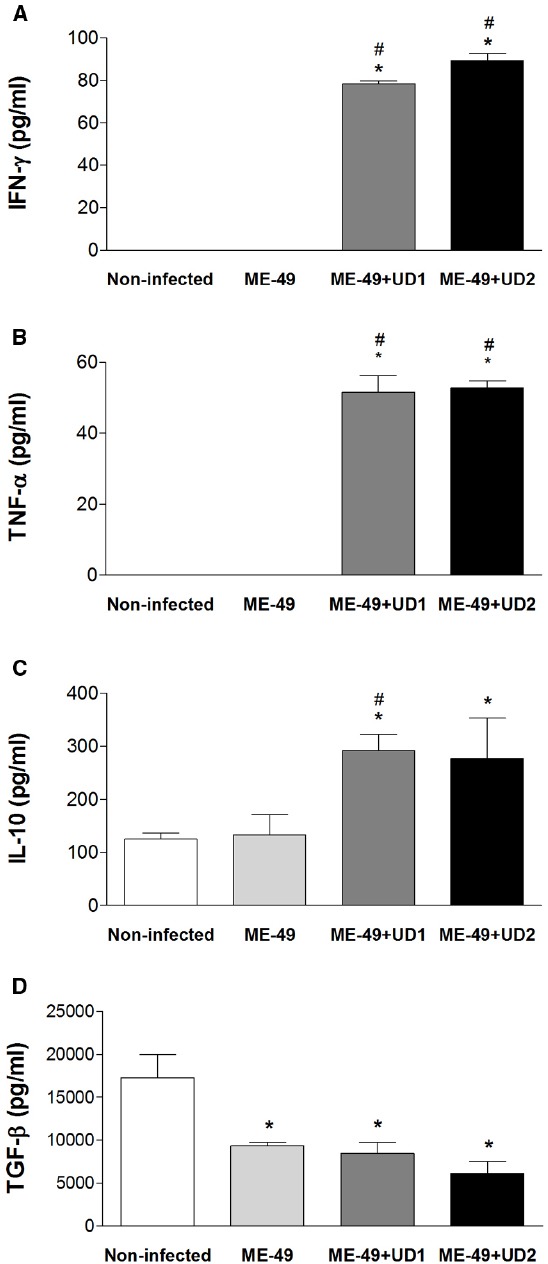
**Production of IFN-γ (A), TNF-α (B), IL-10 (C), and TGF-β (D) in serum samples from non-infected *C. callosus* females, females chronically infected with ME-49 and females chronically infected with ME-49 and reinfected in the first day of pregnancy by TgChBrUD1 (UD1) or TgChBrUD2 (UD2) strains of *Toxoplasma gondii* (n = 7 for each group).** *Comparison between non-infected with ME-49 chronically infected pregnant females, TgChBrUD1 and TgChBrUD2 reinfected pregnant females (Kruskal–Wallis test P < 0.05). #Comparison between ME-49 chronically infected pregnant females with TgChBrUD1 and TgChBrUD2 reinfected pregnant females (Kruskal–Wallis test P < 0.05).

To determine whether the infection with *T. gondii* may change the balance of Th1/Th2 type reactivity in reinfected females, the levels of IFN-γ, TNF-α, IL-10, and TGF-β1 in serum samples from groups were measured. The IFN-γ and TNF-α levels were higher in TgChBrUD1 and TgChBrUD2 reinfected females compared with non-infected and ME-49-chronically infected females (*P* < 0.05; Figures 5A,B). The IL-10 levels were higher in TgChBrUD1 and TgChBrUD2 reinfected females compared with non-infected and were higher in TgChBrUD1 reinfected females compared with ME-49-chronically infected females (*P* < 0.05; Figure 5C). No significant differences were found in IFN-γ, TNF-α, and IL-10 levels between non-infected and ME-49-chronically infected females or between TgChBrUD1 and TgChBrUD2 reinfected females (Figures 5A–C). When analyzing TGF-β1 in serum samples, low levels of the cytokine were observed in ME-49-chronically infected females, TgChBrUD1 and TgChBrUD2 reinfected females compared with non-infected females (*P* < 0.05; Figure 5D).

### ME-49 CHRONICALLY INFECTED PREGNANT FEMALES AND TgChBrUD1 REINFECTED PREGNANT FEMALES DEVELOPED HIGH IgG2a/IgG1 RATIOS

**FIGURE 6:**
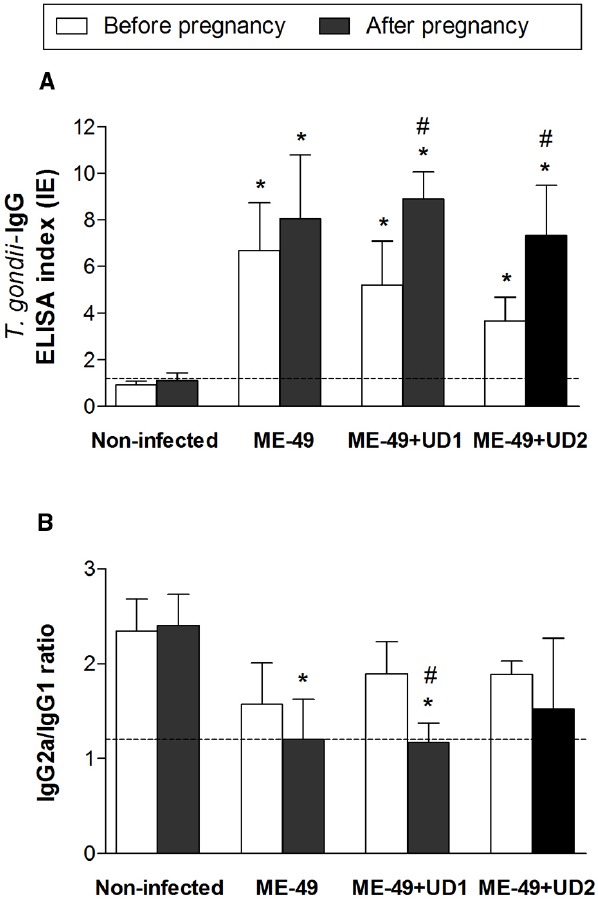
**Levels of *Toxoplasma gondii*-specific total IgG (A) and IgG2a/IgG1 ratio (B) in serum samples from TgChBrUD1 (UD1) and TgChBrUD2 (UD2) reinfected pregnant females (n = 7 for each group) compared with non-infected and ME-49 chronically infected females before and after pregnancy.** Antibody levels were measured by ELISA and expressed as an ELISA index. *Comparison between non-infected or ME-49 chronically infected females with TgChBrUD1 and TgChBrUD2 reinfected females before and after pregnancy (Kruskal-Wallis test P < 0.05). #Comparison between TgChBrUD1 and TgChBrUD2 reinfected pregnant females before and after pregnancy (Kruskal-Wallis test P < 0.05).

In order to additionally verify whether reinfection interferes with the Th1 response induced by *T. gondii*, the specific IgG, IgG1, and IgG2a levels were measured by ELISA in *C. callosus* serum samples. Seroconversion was confirmed before pregnancy, on day 55 of infection with ME-49 strain (Figure 6A). The comparison between IgG levels before pregnancy and after pregnancy showed no significant differences in non-infected females and ME-49 chronically infected females, while TgChBrUD1 and TgChBrUD2 reinfected females showed higher IgG levels after pregnancy (Figure 6A). In addition, no significant differences were found in IgG levels in the serum from ME-49 chronically infected females compared with TgChBrUD1 or TgChBrUD2 reinfected females before or after pregnancy. Also, no significant differences were found when TgChBrUD1 and TgChBrUD2 reinfected females were compared (Figure 6A). The analysis of IgG1 and IgG2a showed low levels for all groups (data not shown). The ratio of IgG2a/IgG1 was lower in ME-49 chronically infected females and TgChBrUD1 reinfected females after pregnancy (Figure 6B). The comparison between IgG2a/IgG1 ratio before pregnancy and after pregnancy showed no significant differences in ME-49 chronically infected females and TgChBrUD2 reinfected females, while TgChBrUD1 reinfected females showed lower IgG2a/IgG1 ratios after pregnancy (Figure 6B).

## DISCUSSION

Congenital toxoplasmosis resulting from chronically infected immunocompetent pregnant women is considered a rare event, being attributed to either reactivation or reinfection (Silveira et al., 2003; Elbez-Rubinstein et al., 2009; Remington et al., 2011). Reinfection cases may be associated with exposure to a large number of parasites, to a more virulent strain or to a parasite of a different genotype (Elbez-Rubinstein et al., 2009; Valdes et al., 2011; Carlier et al., 2012), since the tachyzoites of virulent strains have a greater capacity for invading cells, being able to cross biological barriers and multiplying in particular intracellular compartments (Carlier et al., 2012).

Congenital transmission of *T. gondii* generally occurs only during the acute phase of infection in *C. callosus* (Ferro et al., 2002; Barbosa et al., 2007), similar to what is observed in pregnant women. Our previous studies showed that congenital toxoplasmosis does not occur in females chronically infected with the *T. gondii* ME-49 strain and reinfected with the same strain or reinfected with a virulent *T. gondii* RH strain (Ferro et al., 2002; Franco et al., 2011). In this study, our data demonstrated that *C. callosus* females chronically infected with the ME-49 strain survived after reinfection with the *T. gondii* TgChBrUD1 or TgChBrUD2 strains. Also, it is possible to observe vertical transmission of the parasite, when this host is reinfected with these Brazilian strains.

Previous studies from our group have shown that *C. callosus* is resistant to ME-49 and highly susceptible to infection by RH (Favoreto-Junior et al., 1998). Our recent study showed that *C. callosus* males and females are also highly susceptible to infection by TgChBrUD1 or TgChBrUD2, with mortality after 9 doi (Franco et al., 2014). Recently, a study using mice Swiss females showed that the prime-infection with the ME-49 strain conferred protection against reinfection with the virulent strains CH3 and EGS (Silva et al., 2012). In the present study, we observed that *C. callosus* females chronically infected with ME-49 survived for more than 21 days after TgChBrUD1 or TgChBrUD2 reinfection. Thus, this evidence suggests that primary infection in *C. callosus* elicits an acquired immune response capable of protecting immunocompetent animals against the virulent strain used in the reinfection.

The immunity acquired after primary infection by *T. gondii* has been considered to be efficient in preventing vertical transmission during reinfection (Abou-Bacar et al., 2004; Pfaff et al., 2007). However, cases of congenital toxoplasmosis have been reported in infants born to immunocompetent mothers who had been infected with the parasite before conception, suggesting the occurrence of maternal reinfection during pregnancy (Elbez-Rubinstein et al., 2009). Thus, the hypothesis that the primary *T. gondii* infection leads to life-long immunity and prevents vertical transmission during reinfection has been questioned by several authors using immunocompetent murine models (Ferro et al., 2002; Freyre et al., 2006; Pezerico et al., 2009; Franco et al., 2011). Some studies showed that complete protection against congenital toxoplasmosis is possible when strains belonging to the same genotype (Ferro et al., 2002) or clonal strains (Pezerico et al., 2009; Franco et al., 2011) are used for infection and reinfection. On the other hand, reinfection of chronically infected rats with heterologous and clonal *T. gondii* strains showed a considerable amount of parasites in fetal tissues (Freyre et al., 2006).

Recently, natural mixed infections, resulting from coincident or sequential exposure to parasites of different genotypes, have been observed in humans (Andrade et al., 2010; Carneiro et al., 2013), although it is still unclear whether the protection triggered by the primary infection is genotype-specific. In the present study, we observed vertical transmission of *T. gondii* in *C. callosus* females chronically infected with ME-49 and reinfected with TgChBrUD1 or TgChBrUD2. In addition, our data confirm that vertical transmission of *T. gondii* does not occur in *C. callosus* females chronically infected with ME-49 (Barbosa et al., 2007) despite infection of placental tissues, indicating reactivation of latent infection during gestation and demonstrating the role of the placental barrier in preventing vertical transmission.

Our data showed that when groups of reinfected animals were compared, TgChBrUD2 reinfected females were more susceptible during pregnancy. This group presented lower survival and the reinfection induced a higher morbidity score, showing that when pregnant, females are susceptible to reinfection by Brazilian strains. Moreover, TgChBrUD2 reinfected females presented a high number of pregnant animals with fetal reabsorption and a high fetal loss rate. When the parasitism was analyzed, TgChBrUD2 reinfected females showed higher parasite loads in uterus tissues compared to TgChBrUD1 reinfected females.

Congenital toxoplasmosis may occur due to either an exogenous (reinfection) or an endogenous origin (reactivation; Abou-Bacar et al., 2004; Pfaff et al., 2007). Besides evaluating the *T. gondii* DNA in tissues from reinfected females, we also analyzed the participation of the *T. gondii* genotype in the process of reinfection. The genotyping showed the ME-49 or TgChBrUD1 strains in uterus tissues from TgChBrUD1 reinfected females but only the TgChBrUD2 strain in uterus tissues from TgChBrUD2 reinfected females. When placentas and fetuses were analyzed, it was observed that TgChBrUD1 reinfected females presented parasites in both locations. Also, the only pregnant female reinfected with TgChBrUD2 presented parasites in both placenta and fetal tissue as well. Infection was confirmed by PCR of brain tissue from mouse bioassays. Our results showed ME-49 or TgChBrUD1 in placental tissues and TgChBrUD1 strain in fetal tissues from TgChBrUD1 reinfected females, while only TgChBrUD2 was found in placentas from TgChBrUD2 reinfected females. Therefore, reinfection with these Brazilian strains promoted vertical transmission of the *T. gondii* ME-49, TgChBrUD1 or TgChBrUD2 strains in *C. callosus* females.

The genotyping results for TgChBrUD2 reinfected females showed only one type of strain in tissue samples from reinfected females, though this does not exclude the possibility of the presence of ME-49, since a low DNA concentration can be undetectable by RFLP–PCR. It is also necessary to address divergent results observed in tissues from the same females in different assays. This may be explained by the fact that we used different fetuses from the same mother to evaluate vertical transmission in each of the assays (immunohistochemistry, mice bioassay, and PCR assay). For example, the fetuses from one female used in bioassays were not infected and, for this reason, the Swiss mice remained seronegative and survived until 30 days after infection. On the other hand, fetuses from different females of the same group were infected and were responsible for the death of Swiss mice around 7 days after infection or responsible for the PCR positive results in brains of these animals. We found the Swiss mice dead around 7 days after infection, suggesting that these animals died because of *T. gondii* acute infection. Therefore, it is important to take into account that these different results found in different approaches could also be explained by biological differences in terms of site of implantations in the uterus, resulting in particular microenvironments facilitating or not facilitating the infection by *T. gondii*.

The TgChBrUD1 and TgChBrUD2 strains belong to the non-clonal genotype profile (type BrII and type BrI, respectively) predominant in Brazil, while the ME-49 strain belongs to the clonal type II genotype, frequently found in Europe and in North America (Pena et al., 2008). These stark differences are likely to be due to the predominance of more virulent genotypes of the parasite in Brazil, which are rarely found in Europe. Thus, our results corroborate the authors’ hypothesis that the immunity acquired against European strains may not protect against reinfection by strains of a different genotype (Freyre et al., 2006; Elbez-Rubinstein et al., 2009).

Because these strains are drawing on genes that are outside the domestic gene pool that contributes to types I, II, and III, the severity of infection could be explained, in part, by a poor adaptation of the immune system against these genotypes of *T. gondii*. Host control of *Toxoplasma* induces potent Th1-type immune responses with the production of the pro-inflammatory cytokine IL-12, which is produced by macrophages and DCs in response to Toll-like receptor (TLR) recognition of molecular structures broadly conserved across microbial species (Yarovinsky, 2014). IL-12 in turn activates NK and T cells to secrete IFN-γ, which plays a major role in restricting proliferation of tachyzoites during the acute stage of infection (Abou-Bacar et al., 2004; Melo et al., 2011; Suzuki et al., 2011). The latter activates effector mechanisms for intracellular elimination of *Toxoplasma*, including the activation of interferon-regulated GTPases, induction of reactive nitrogen intermediates, tryptophan degradation and autophagy in human cells (Melo et al., 2011; Gazzinelli et al., 2014).

Acute *T. gondii* infection during gestation may disrupt the maternal-fetal immunological balance in favor of anti-parasitic pro-inflammatory abortogenic cytokines, such as IFN-γ and TNF-α, which are reported to be potentially deleterious for conception (Shiono et al., 2007). In this study, *C. callosus* females chronically infected with ME-49 and reinfected with TgChBrUD1 or TgChBrUD2 showed high levels of IFN-γ, TNF-α, and IL-10 compared with ME-49 chronically infected females. On the other hand, low levels of TGF-β were observed in ME-49 chronically infected females and TgChBrUD1 and TgChBrUD2 reinfected females compared with non-infected females. These data suggest that the elevated inflammatory immune response induced by *T. gondii* could be involved in the higher number of reabsorbed fetuses and absorbed implantation sites observed in females reinfected with Brazilian strains. Moreover, even the strong pro-inflammatory immune response induced by the parasite was not sufficient to control the infection, being unable to prevent the vertical transmission and the high parasite concentration observed in placenta, liver, spleen and lung tissue of reinfected females.

Accordingly, a previous study showed high levels of IFN-γ when C57BL/6 mice were infected with RH strain in an early stage of pregnancy and this phenomenon was related to high reabsorption rates of implantation sites (Ge et al., 2008). The high levels of IFN-γ produced suggested that the Th1 type cellular immune response was mainly activated. On the other hand, an increase in IL-10 was also observed, suggesting that anti-inflammatory responses were activated.

Both cellular and humoral components of the immune system play a role in resistance against secondary infection. Reinfection is accompanied by an intense immune response, often manifested by the elevation of IgG levels and the appearance of IgM antibodies (Gavinet et al., 1997). In the present study, higher IgG levels were observed in serum from TgChBrUD1 and TgChBrUD2 reinfected pregnant females after pregnancy compared with IgG levels before pregnancy, confirming the ability of *T. gondii* to induce a humoral immune response after reinfection. The ME-49 chronically infected females and TgChBrUD1 reinfected females presented lower IgG2a/IgG1 ratio in pregnancy compared with IgG2a/IgG1 ratio before pregnancy, suggesting the establishment of a Th2 type immune response in pregnancy and reinfection. The Th2 pattern is characterized by a preferential production of complement-independent IgG1 antibodies, whereas Th1 responses are characterized by production of complement-dependent IgG2a antibodies. Consistent with these isotype profiles, antigen-specific CD4^+^ Th2 type T helper cells produce IL-4 that supports switching to IgG1, while Th1 type T helper cells are characterized by the generation of IFN-γ that supports switching to IgG2a (Mosmann et al., 1997). Thus, our data indicate that the immune response developed during gestation and reinfection in TgChBrUD1 reinfected females could be associated with higher pro-inflammatory cytokines and IgG1 subtype profiles showing a predominance of mixed Th1/Th2 responses. On the other hand, TgChBrUD2 reinfected females presented higher pro-inflammatory cytokines and IgG2a subtype profiles, suggesting predominance of the Th1 response.

In conclusion, our results showed that *C. callosus* females chronically infected by a *T. gondii* classical type II clonal strain survive after reinfection with Brazilian strains, but the acquired immune response of this host is insufficient to prevent congenital toxoplasmosis. The pregnancy promoted *T. gondii* ME-49 strain reactivation and the reinfection caused vertical transmission of *T. gondii*. Also, pregnant females that have been reinfected by Brazilian strains developed strong pro-inflammatory immune responses including Th1 cytokines and antibody isotype, leading to damage for the developing fetuses.

### Conflict of Interest Statement

The Reviewer Solange Maria Gennari declares that, despite having collaborated with authors E. K. Shwab and Chunlei Su, the review process was handled objectively and no conflict of interest exists. The authors declare that the research was conducted in the absence of any commercial or financial relationships that could be construed as a potential conflict of interest.

## References

[B1] Abou-Bacar A. Pfaff A. W. Letscher-Bru V. Filisetti D. Rajapakse R. Antoni E. (2004). Role of gamma interferon and T cells in congenital *Toxoplasma* transmission. Parasite Immunol. 26, 315–318. 10.1111/j.0141-9838.2004.00713.x 15679627

[B2] Adams Waldorf K. M. McAdams R. M. (2013). Influence of infection during pregnancy on fetal development. Reproduction 146, R151–R162. 10.1530/REP-13-0232 23884862 PMC4060827

[B3] Andrade G. M. Vasconcelos-Santos D. V. Carellos E. V. Romanelli R. M. Vitor R. W. Carneiro A. C. (2010). Congenital toxoplasmosis from a chronically infected woman with reactivation of retinochoroiditis during pregnancy. J. Pediatr. (Rio. J.) 86, 85–88 10.2223/JPED.1948 19918624

[B4] Barbosa B. F. Silva D. A. Costa I. N. Pena J. D. Mineo J. R. Ferro E. A. (2007). Susceptibility to vertical transmission of *Toxoplasma gondii* is temporally dependent on the preconceptional infection in *Calomys callosus*. Placenta 28, 624–630. 10.1016/j.placenta.2006.10.011 17182099

[B5] Bartley P. M. Wright S. Sales J. Chianini F. Buxton D. Innes E. A. (2006). Long-term passage of tachyzoites in tissue culture can attenuate virulence of *Neospora caninum in vivo*. Parasitology 133, 421–432. 10.1017/S0031182006000539 16762097

[B6] Bojar I. Szymanska J. (2010). Environmental exposure of pregnant women to infection with *Toxoplasma gondii*—state of the art. Ann. Agric. Environ. Med. 17, 209–214. 21186761

[B7] Carlier Y. Truyens C. Deloron P. Peyron F. (2012). Congenital parasitic infections: a review. Acta Trop. 121, 55–70. 10.1016/j.actatropica.2011.10.018 22085916

[B8] Carneiro A. C. Andrade G. M. Costa J. G. Pinheiro B. V. Vasconcelos-Santos D. V. Ferreira A. M. (2013). Genetic characterization of *Toxoplasma gondii* revealed highly diverse genotypes for isolates from newborns with congenital toxoplasmosis in southeastern Brazil. J. Clin. Microbiol. 51, 901–907. 10.1128/JCM.02502-12 23284022 PMC3592078

[B9] Dubey J. P. Lago E. G. Gennari S. M. Su C. Jones J. L. (2012). Toxoplasmosis in humans and animals in Brazil: high prevalence, high burden of disease, and epidemiology. Parasitology 139, 1375–1424. 10.1017/S0031182012000765 22776427

[B10] Elbez-Rubinstein A. Ajzenberg D. Darde M. L. Cohen R. Dumetre A. Yera H. (2009). Congenital toxoplasmosis and reinfection during pregnancy: case report, strain characterization, experimental model of reinfection, and review. J. Infect. Dis. 199, 280–285. 10.1086/595793 19032062

[B11] Favoreto-Junior S. Ferro E. A. Clemente D. Silva D. A. Mineo J. R. (1998). Experimental infection of *Calomys callosus* (Rodentia, Cricetidae) by *Toxoplasma gondii*. Mem. Inst. Oswaldo Cruz 93, 103–107. 10.1590/S0074-02761998000100018 9698850

[B12] Ferro E. A. Silva D. A. Bevilacqua E. Mineo J. R. (2002). Effect of *Toxoplasma gondii* infection kinetics on trophoblast cell population in *Calomys callosus*, a model of congenital toxoplasmosis. Infect. Immun. 70, 7089–7094. 10.1128/IAI.70.12.7089-7094.2002 12438390 PMC133059

[B13] Franco P. S. Ribeiro M. Lopes-Maria J. B. Costa L. F. Silva D. A. De Freitas Barbosa B. (2014). Experimental infection of *Calomys callosus* with atypical strains of *Toxoplasma gondii* shows gender differences in severity of infection. Parasitol. Res. 113, 2655–2664. 10.1007/s00436-014-3920-y 24781027

[B14] Franco P. S. Silva D. A. Costa I. N. Gomes A. O. Silva A. L. Pena J. D. (2011). Evaluation of vertical transmission of *Toxoplasma gondii* in *Calomys callosus* model after reinfection with heterologous and virulent strain. Placenta 32, 116–120. 10.1016/j.placenta.2010.11.012 21146211

[B15] Freyre A. Falcon J. Mendez J. Rodriguez A. Correa L. Gonzalez M. (2006). *Toxoplasma gondii*: partial cross-protection among several strains of the parasite against congenital transmission in a rat model. Exp. Parasitol. 112, 8–12. 10.1016/j.exppara.2005.08.009 16202411

[B16] Gavinet M. F. Robert F. Firtion G. Delouvrier E. Hennequin C. Maurin J. R. (1997). Congenital toxoplasmosis due to maternal reinfection during pregnancy. J. Clin. Microbiol. 35, 1276–1277. 9114425 10.1128/jcm.35.5.1276-1277.1997 PMC232747

[B17] Gazzinelli R. T. Mendonca-Neto R. Lilue J. Howard J. Sher A. (2014). Innate resistance against *Toxoplasma gondii*: an evolutionary tale of mice, cats, and men. Cell Host Microbe 15, 132–138. 10.1016/j.chom.2014.01.004 24528860 PMC4006104

[B18] Ge Y. Y. Zhang L. Zhang G. Wu J. P. Tan M. J. Hu E. (2008). In pregnant mice, the infection of *Toxoplasma gondii* causes the decrease of CD4^+^CD25^+^ -regulatory T cells. Parasite Immunol. 30, 471–481. 10.1111/j.1365-3024.2008.01044.x 18627509

[B19] Gilbert R. E. Freeman K. Lago E. G. Bahia-Oliveira L. M. Tan H. K. Wallon M. (2008). Ocular sequelae of congenital toxoplasmosis in Brazil compared with Europe. PLoS Negl. Trop. Dis. 2:e277. 10.1371/journal.pntd.0000277 18698419 PMC2493041

[B20] Hill R. D. Gouffon J. S. Saxton A. M. Su C. (2012). Differential gene expression in mice infected with distinct *Toxoplasma* strains. Infect. Immun. 80, 968–974. 10.1128/IAI.05421-11 22144491 PMC3294647

[B21] Howe D. K. Sibley L. D. (1995). *Toxoplasma gondii* comprises three clonal lineages: correlation of parasite genotype with human disease. J. Infect. Dis. 172, 1561–1566. 10.1093/infdis/172.6.1561 7594717

[B22] Joachim R. A. Hildebrandt M. Oder J. Klapp B. F. Arck P. C. (2001). Murine stress-triggered abortion is mediated by increase of CD8^+^ TNF-α^+^ decidual cells via substance P. Am. J. Reprod. Immunol. 45, 303–309. 10.1111/j.8755-8920.2001.450506.x 11432405

[B23] Khan A. Taylor S. Ajioka J. W. Rosenthal B. M. Sibley L. D. (2009). Selection at a single locus leads to widespread expansion of *Toxoplasma gondii* lineages that are virulent in mice. PLoS Genet. 5:e1000404. 10.1371/journal.pgen.1000404 19266027 PMC2644818

[B24] Kusakabe K. Naka M. Ito Y. Eid N. Otsuki Y. (2008). Regulation of natural-killer cell cytotoxicity and enhancement of complement factors in the spontaneously aborted mouse placenta. Fertil. Steril. 90, 1451–1459. 10.1016/j.fertnstert.2007.07.1331 18068164

[B25] Lowry O. H. Rosebrough N. J. Farr A. L. Randall R. J. (1951). Protein measurement with the Folin phenol reagent. J. Biol. Chem. 193, 265e75. 14907713

[B26] Melo M. B. Jensen K. D. Saeij J. P. (2011). *Toxoplasma gondii* effectors are master regulators of the inflammatory response. Trends Parasitol. 27, 487–495. 10.1016/j.pt.2011.08.001 21893432 PMC3200456

[B27] Mineo J. R. Camargo M. E. Ferreira A. W. (1980). Enzyme-linked immunosorbent assay for antibodies to *Toxoplasma gondii* polysaccharides in human toxoplasmosis. Infect. Immun. 27, 283e7. 7380534 10.1128/iai.27.2.283-287.1980 PMC550761

[B28] Mosmann T. R. Li L. Hengartner H. Kagi D. Fu W. Sad S. (1997). Differentiation and functions of T cell subsets. Ciba Found. Symp. 204, 148–154; discussion 154–148. 9107418 10.1002/9780470515280.ch10

[B29] Pena H. F. Gennari S. M. Dubey J. P. Su C. (2008). Population structure and mouse-virulence of *Toxoplasma gondii* in Brazil. Int. J. Parasitol. 38, 561–569. 10.1016/j.ijpara.2007.09.004 17963770

[B30] Pezerico S. B. Langoni H. Da Silva A. V. Da Silva R. C. (2009). Evaluation of *Toxoplasma gondii* placental transmission in BALB/c mice model. Exp. Parasitol. 123, 168–172. 10.1016/j.exppara.2009.06.015 19563804

[B31] Pfaff A. W. Abou-Bacar A. Letscher-Bru V. Villard O. Senegas A. Mousli M. (2007). Cellular and molecular physiopathology of congenital toxoplasmosis: the dual role of IFN-γ. Parasitology 134, 1895–1902. 10.1017/S0031182007000200 17958925

[B32] Remington J. S. Mcleod R. Wilson C. B. Desmonts G. (2011). “Toxoplasmosis,” in Infectious Diseases of the Fetus and Newborn Infant, 7th Edn, eds Remington J. S. Klein J. O. Wilson C. B. Nizet V. Maldonado Y. A. (Philadelphia: Elsevier Saunders), 918–1041.

[B33] Robert-Gangneux F. Murat J. B. Fricker-Hidalgo H. Brenier-Pinchart M. P. Gangneux J. P. Pelloux H. (2011). The placenta: a main role in congenital toxoplasmosis? Trends Parasitol. 27, 530–536. 10.1016/j.pt.2011.09.005 22079164

[B34] Saeij J. P. Boyle J. P. Coller S. Taylor S. Sibley L. D. Brooke-Powell E. T. (2006). Polymorphic secreted kinases are key virulence factors in toxoplasmosis. Science 314, 1780–1783. 10.1126/science.1133690 17170306 PMC2646183

[B35] Schlüter D. Däubener W. Schares G. Groß U. Pleyer U. Lüder C. (2014). Animals are key to human toxoplasmosis. Int. J. Med. Microbiol. 7, 917–929. 10.1016/j.ijmm.2014.09.002 25240467

[B36] Shiono Y. Mun H. S. He N. Nakazaki Y. Fang H. Furuya M. (2007). Maternal-fetal transmission of *Toxoplasma gondii* in interferon-gamma deficient pregnant mice. Parasitol. Int. 56, 141–148. 10.1016/j.parint.2007.01.008 17307382

[B37] Shwab E. K. Zhu X. Q. Majumdar D. Pena H. F. Gennari S. M. Dubey J. P. (2014). Geographical patterns of *Toxoplasma gondii* genetic diversity revealed by multilocus PCR-RFLP genotyping. Parasitology 141, 453–461. 10.1017/S0031182013001844 24477076

[B38] Silva L. A. Brandao G. P. Pinheiro B. V. Vitor R. W. (2012). Immunosuppression with cyclophosphamide favors reinfection with recombinant *Toxoplasma gondii* strains. Parasite 19, 249–257. 10.1051/parasite/2012193249 22910667 PMC3671442

[B39] Silveira C. Ferreira R. Muccioli C. Nussenblatt R. Belfort R. Jr. (2003). Toxoplasmosis transmitted to a newborn from the mother infected 20 years earlier. Am. J. Ophthalmol. 136, 370–371 10.1016/S0002-9394(03)00191-0 12888070

[B40] Su C. Khan A. Zhou P. Majumdar D. Ajzenberg D. Darde M. L. (2012). Globally diverse *Toxoplasma gondii* isolates comprise six major clades originating from a small number of distinct ancestral lineages. Proc. Natl. Acad. Sci. U.S.A. 109, 5844–5849. 10.1073/pnas.1203190109 22431627 PMC3326454

[B41] Su C. Shwab E. K. Zhou P. Zhu X. Q. Dubey J. P. (2010). Moving towards an integrated approach to molecular detection and identification of *Toxoplasma gondii*. Parasitology 137, 1–11. 10.1017/S0031182009991065 19765337

[B42] Sullivan W. J. Jr. Jeffers V. (2012). Mechanisms of *Toxoplasma gondii* persistence and latency. FEMS Microbiol. Rev. 36, 717–733. 10.1111/j.1574-6976.2011.00305.x 22091606 PMC3319474

[B43] Suzuki Y. Sa Q. Gehman M. Ochiai E. (2011). Interferon-gamma- and perforin-mediated immune responses for resistance against *Toxoplasma gondii* in the brain. Expert Rev. Mol. Med. 13, e31. 10.1017/S1462399411002018 22005272 PMC3372998

[B44] Valdes V. Legagneur H. Watrin V. Paris L. Hascoet J. M. (2011). *Toxoplasmose congénitale secondaire à une réinfection maternelle pendant la grossesse* [Congenital toxoplasmosis due to maternal reinfection during pregnancy]. Arch. Pediatr. 18, 761–763. 10.1016/j.arcped.2011.04.011 21600743

[B45] Watson E. D. Cross J. C. (2005). Development of structures and transport functions in the mouse placenta. Physiology 20, 180–193. 10.1152/physiol.00001.2005 15888575

[B46] Wujcicka W. Wilczynski J. Nowakowska D. (2014). Do the placental barrier, parasite genotype and Toll-like receptor polymorphisms contribute to the course of primary infection with various *Toxoplasma gondii* genotypes in pregnant women? Eur. J. Clin. Microbiol. Infect. Dis. 33, 703–709. 10.1007/s10096-013-2017-3 24292064 PMC3996274

[B47] Yarovinsky F. (2014). Innate immunity to *Toxoplasma gondii* infection. Nat. Rev. Immunol. 14, 109–121. 10.1038/nri3598 24457485

[B48] Zenclussen A. C. Joachim R. Hagen E. Peiser C. Klapp B. F. Arck P. C. (2002). Heme oxygenase is downregulated in stress-triggered and interleukin-12-mediated murine abortion. Scand. J. Immunol. 55, 560–569. 10.1046/j.1365-3083.2002.01091.x 12028558

